# Gene therapy in neuromuscular disorders

**DOI:** 10.1590/0004-282X-ANP-2022-S135

**Published:** 2022-08-12

**Authors:** Rodrigo Holanda Mendonça, Edmar Zanoteli

**Affiliations:** 1 Universidade de São Paulo, Faculdade de Medicina, Hospital das Clínicas, Departamento de Neurologia, São Paulo, SP, Brazil.

**Keywords:** Genetic Therapy, Dependovirus or Adeno-Associated Virus (AAVs), Muscular Atrophy, Spinal, Survival of Motor Neuron 1 Protein, Genetic Vectors, Terapia Genética, Dependovirus ou Virus Adeno-Associados (AAVs), Atrofia Muscular Espinal, Proteína 1 de Sobrevivência do Neurônio Motor, Vetores Genéticos

## Abstract

Monogenic neuromuscular disorders are potentially treatable through gene therapy. Using viral vectors, a therapeutic transgene aims to restore normal levels of a protein not produced by the defective gene, or to silence a gene whose expression leads to toxic effects. Spinal Muscular Atrophy (SMA) is a good example of a monogenic disease that currently has an AAV9-based vector gene therapy as a therapeutic option. In this review, we intend to discuss the viral vectors and their mechanisms of action, in addition to reviewing the clinical trials that supported the approval of gene therapy (AVXS-101) for SMA as well as neuromuscular diseases that are potentially treatable with gene replacement therapy.

## Resumo

Doenças neuromusculares monogênicas são potencialmente tratáveis através de terapia gênica. Utilizando-se de vetores virais, um transgene terapêutico objetiva repor os níveis normais de uma proteina não produzida pelo gene defeituoso ou silenciar um gene cuja expressão leva a efeitos tóxicos. A Atrofia Muscular Espinhal (AME) é um bom exemplo de doença monogenica que atualmente tem uma terapia gênica com vetor viral AAV9 como opção terapêutica. Nesta revisão, pretendemos discutir os vetores virais e macanismos de ação utilizados, além de revisar os ensaios clínicos que embasaram a aprovação da terapia gênica (AVXS-101) para AME, assim como doenças neuromusculares potencialmente tratáveis com terapia de reposição gênica.

## INTRODUCTION

Several neuromuscular disorders, both those that affect the motor neurons, such as Spinal Muscular Atrophy (SMA), and others like muscular dystrophies such as Duchenne muscular dystrophy (*DMD*), are fatal monogenic diseases caused by loss-of-function mutations. They are therefore potential targets for gene replacement therapy, in which the function of a defective gene can be replaced by a functional transgene through the use of a viral vector^1^. 

There have been several attempts to transpose gene therapy from translational medicine into clinical practice in the last 40 years^2^ and, more recently, the approval of the first gene therapy for the treatment of a neuromuscular disease took place^3^. Gene therapy is now a therapeutic option for SMA, using an adeno-associated virus 9 (AAV9) as a vector. 

Before addressing this specific therapy, we will review the different mechanisms by which a gene therapy can treat a monogenic disease and the types of viral vector platforms that can be used. Later in the text we also review other potential neuromuscular diseases candidates for gene therapy.

## MECHANISM OF ACTION

The most usual mechanism by which gene therapy acts is the replacement of a defective gene through a functional transgene^1^. SMA is a good example that can be treated in this way. The disease is caused by deletions in the *SMN1* (survival motor neuron) gene^4^. This common deletion leads to low levels of SMN protein and to death of the motor neurons in the anterior horn of the spinal cord and brainstem motor nuclei. To restore normal levels of SMN protein, a transgene that is a copy of the human *SMN1* gene, is transfected into patients with the disease, and begins to transcribe functional SMN protein, avoiding the natural course of the disease^3^. 

Another mechanism of action of a gene therapy is also to silence an overexpressed gene that generates a disease. This example can be understood through the familial Amyotrophic Lateral Sclerosis (ALS) caused by mutations in the Superoxide Dismutase 1 (*SOD1*) gene. Mutations in *SOD1* are believed to determine ALS through toxic gain of function caused by aggregation of misfolded SOD1 protein^5^. It is possible to silence the expression of the *SOD1* gene with the use of an adeno-associated vírus sorotype rh10 containing an anti-*SOD1* microRNA (AAV-miR-*SOD1*), which leads to degradation of *SOD1* messenger RNA^6^ ^,^ ^7^.

## VIRAL VECTORS

In vivo gene therapy entails the direct administration of a vector carrying a therapeutic transgene into the patient^8^. Viral vectors are naturally occurring viruses that have been modified by replacing the viral gene with a desired transgene^9^. The removal of viral genes means that this modified virus does not replicate or trigger the same immune response as the wild-type virus^10^. In humans, viral vectors have shown success achieved by in vivo delivery of the therapeutic gene into the patient by vectors based on retroviruses, adenoviruses (Ads) or adeno-associated viruses (AAVs)^11^.

Lentiviruses, such as those from the *Retroviridae* family, have their DNA integrated into the host genome in a non-random manner with preference for transcriptionally active sites and their use in clinical practice remains a challenge^12^. Ad vectors have the advantages of high transduction efficiency, do not integrate into host DNA and persist as an episome, while they have a broad tropism for different tissue targets. Their major drawback persists in widely pre-existing viral immunity among the general population and the consequent life-threatening strong innate immune responses to the capsid proteins^13^.

The AAV vectors are small vectors with huge potential, now used in clinical practice. The success of the use of this viral vector is due to the wide-ranging tropism profiles, targeting the central nervous system, the eyes, liver, heart, and muscle. Also, AAVs are accepted as least immunogenic, with much less vector-related toxicity when compared to Ad vectors^14^. Like Ad vectors, AAV vectors do not integrate into the host genome, persisting instead as an episomal DNA, without oncogenic risk.

## VIRAL VECTOR DESIGN

**Figure 1.**
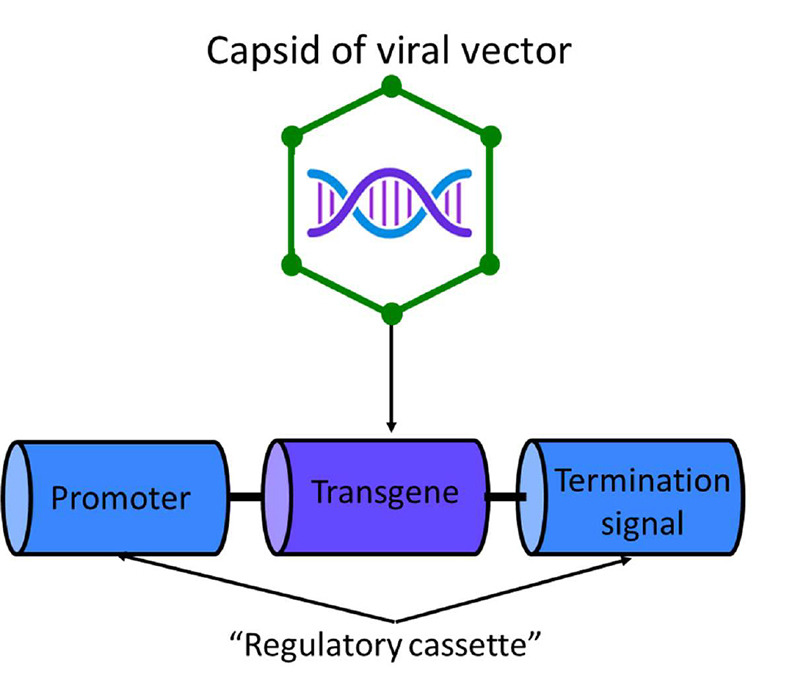
Main components of a gene therapy (in parentheses, AVXS-101 components are specified): Capsid of viral vector (scAAV9); therapeutic transgene (Human *SMN1*, deliver a copy of the gene encoding the SMN protein); and the “regulatory cassette,” the enhancer or promoter (Hybrid CMV enhancer and chicken beta-actin promoter activate the transgene to allow continuous and sustained expression of the *SMN1* protein) and the termination signal or inverted terminal repeats (ITRs) (Self-complementary AAV ITRs increase the rate at which the double-stranded transgene is transcribed and the resulting protein is produced). Figure adapted from Wang D, Gao G. 2014^15^

Three of the following are the main components of a viral vector (figure 1): (1) capsid, which is mainly protein-based, and therefore immunogenic. It protects and carries the viral genome, and defines the vector's tissue or cell tropism; (2) the transgene of interest, when expressed in targeted cells, serves to confer a desired effect, either replacing the function of a defective gene or silencing a gene with a toxic effect; and (3) the “regulatory cassette,” the combined enhancer or promoter elements that control stable or transient somatic expression of the transgene as an episome or as a chromosomal integrant^11^ ^,^ ^15^. Also, the expression “cassette” usually includes a termination signal for gene transcription or inverted terminal repeats (ITRs) at either end of the cassette to allow for the synthesis of complementary DNA^16^. As an example of the regulatory cassette, the recombinant vector AAV9 containing a copy of the human *SMN1* gene (AVXS-101) has a combination of a self-complementary feature with a hybrid cytomegalovirus enhancer-chicken beta-actin promoter that enables rapid and sustained expression of SMN protein levels^17^. 

## GENE THERAPY FOR SPINAL MUSCULAR ATROPHY

The potential of gene therapy in SMA was raised when a one-time intravenous administration of a self-complementary adeno-associated viral serotype 9 (scAAV9) that delivered a copy of *SMN1* induced SMN expression in motor neurons and peripheral tissues, and extended the average survival in a murine model of SMA^18^. Other current therapies for the treatment of SMA, such as the use of intrathecal antisense oligonucleotides (ASO), are site-specific for the central nervous system^19^, therefore have an effect on compartmentalized SMN protein levels and require repeated administration every four months^20^. In addition to being administered in a single dose, systemic administration of gene therapy may be advantageous, as SMN protein is ubiquitously expressed and SMA1 affects multiple systems, such as autonomic and enteric nervous systems, and the cardiovascular system^21^. 

After the results of the trial START (formerly known as AVXS-101-CL-101), a Phase 1 study^17^, gene therapy was approved by the Food and Drug Administration (FDA) in 2019, with the name onasemnogene aberpavovec-xioi , with indication for the treatment of SMA in patients up to two years of age^22^. 

## CLINICAL TRIALS

Published or ongoing studies of AVXS-101 are open-label, uncontrolled, and non-randomised (Table 1). The main reason for not including a control group is the fact that there is already an effective drug on the market for the treatment of patients with type 1 SMA^23^. Thus, the clinical trials results with gene therapy are compared with published data on the natural history of the disease^24^ ^,^ ^25^. However, there are important limitations to this methodology, such as the lack of randomization and the difference in baseline characteristics of the groups of patients being compared. It is of note that the effects of gene therapy have not yet been studied in patients in advanced stages of SMA, with severe motor impairment or on mechanical ventilation, making it impossible to extrapolate the results to this group of patients^26^. 

**Table 1.** Main aspects of clinical studies with gene therapy (onasemnogene aberpavovec/AVXS-101) for SMA.

Study/ Identifier in clinicaltrials.gov/ Date started	Phase study and Description	Target population and inclusion criteria	Sample size	Endpoints and results (when available)
AVXS-101-CL-101/ NCT02122952/ 2014	Phase 1, Open-Label, two experimental arms (low and high dose) of IV single-dose AVXS-101	SMA type 1 with two copies of *SMN2* up to six or nine months age	N=15 (3 in low dose cohort and 12 in high dose cohort)	Safety and tolerability/ All patients in high dose (N=12) were event-free* and 10/12 sitting
START/ NCT03421977/2017	Observational, long-term follow-up	Patients from AVXS-101-CL-101 gene replacement therapy clinical trial for SMA Type 1	N=13 (10 patients from previous high cohort)	Long term safety data / 6/10 pt sitting, 2/10 pt standing, 2/10 pt walking in 5 years follow-up- ONGOING
AVXS-101-CL-303 or STR1VE-US/ NCT03306277/ 2017	Phase 3, Open-Label, Single-Arm, of IV single-dose AVXS-101	SMA type 1 with one or two copies of *SMN2* up to 6 months of age	N=22	13 pt (59%) sitting for 30 sec up to 18m and 90.9% of event-free* survival
STR1VE-EU/ NCT03461289/ 2018	Phase 3, Open-Label, Single-Arm, of IV single-dose AVXS-101	SMA type 1 with one or two copies of *SMN2* up to 6 months of age	N=33	14 pt (44%) sitting for 10 sec up to 18m / 31 pt (97%) are event-free* at 14m of age
SPR1NT/ NCT03505099/ 2018	Phase 3, Open-Label, Single-Arm, of IV single-dose AVXS-101	Pre-symptomatic patients with two or three copies of *SMN2* up to 42 days (6 weeks) of live at dosing date	N=30 (cohort 1: two copies of *SMN2* and cohort 2: three copies of *SMN2*)	All pt event-free* at 14m of age and 11/15 (79%) sitting for 30 Sec (cohort 1)/ 8/15 pt standing for 3 sec and 6/15 walking (cohort 2)
STRONG/ NCT03381729/2017	Phase 1, Open-Label, dose-compared study of intrathecal single-dose AVXS-101	SMA type 2, with three copies of *SMN2*, older than 6m and up to 5 years old	N=33 (3 different experimental cohorts: dose A, B and C)	ONGOING - determine optimal dose, standing ability and change in HFMSE/ walking ability and change in ventilation support
OFELIA/ NCT05073133/ 2021	Phase 4, Open-label, single-arm of IV single-dose AVXS-101	Symptomatic SMA (any type) with any copy of *SMN2* up to 24m of age and Weight ≤17 kg.	N=16	ONGOING - AEs and SAEs, changes in vital signs, cardiac safety, and laboratory results/ development of motor milestones

*Death or continuous ventilatory support; AE: adverse events; HFMSE: Hammersmith Functional Motor Scale-Expanded; m: months; IV: intravenous; N: number; pt: patients; SAE: severe adverse events; Sec: seconds; SMA: Spinal Muscular Atrophy; *SMN2:* Survival Motor Neuron gene 2.

Long-term effects of therapy are little known. Thus, any adverse events should be monitored during the course of the disease. Therapy action time is also not known. Studies with a small number of cases have shown that the effect of the therapy has been maintained for at least five years^27^. There are also no studies showing the effects of using more than one dose of therapy. 

FDA approval of gene therapy for SMA was based on clinical studies with SMA patients younger than six months of age. Additional data on patients aged up to two years and weighing up to 13.5 kg are disseminated through presentations at Medical Congresses. This data comes primarily from non-systematic data collection in the United States, where AVXS-101 is approved up to age two. When administered after six months of age and/or in advanced stages of the disease, parents and physicians should be aware that data on efficacy and safety in this group of patients is scarce. In this patient population, it is particularly important for clinicians to discuss the benefits and risks and carefully manage the expectations of parents or patients. Such a recommendation is included in a consensus on treatment with gene therapy for SMA type 1 recently published by European Medical Specialists^28^. 

### AVXS-101-CL-101 AND START TRIAL

Phase 1 study was with single-dose intravenous administration of scAAV9 (*SMN1*) in 15 children with SMA type 1 from 0.9 to 7.9 months of age^17^. At the end of the study, 24 months after the dose, all treated patients in the high dose cohort (n=12) were event-free, that is, they survived without mechanical ventilation. At 24 months, 10/12 were able to sit unsupported for at least 10 seconds, nine were able to sit unsupported for at least 30 seconds, and two were able to stand and walk unaided. Ten of 12 patients in the CL-101 study continue to be followed in a long-term study (5.7 years after administration) and all maintained previously achieved milestones or even reached new milestones, including unsupported sitting, standing with assistance and walking alone^27^. 

### AVXS-101-CL-303/ STR1VE-US

This is a phase 3, open-label, single-arm, single-dose intravenous study of AVXS-101. Twenty-two children with SMA type 1, aged between 0.5 and 5.9 months at administration, were included^29^. Of the 22 patients enrolled, three discontinued the study and two experienced an event (death or permanent ventilation) that led to 90.9% event-free survival (alive without permanent ventilation) at 14 months of age (versus 26% in the untreated PNCR cohort). Thirteen patients (59%) achieved the ability to sit for at least 30 seconds at an average of 18 months; 21 patients (95.5%) achieved a CHOP-INTEND (Children’s Hospital Of Philadelphia Infant Test Of Neuromuscular Disorders) score greater than or equal to 40, 14 (64%) greater than or equal to 50, and five (23%) greater than or equal to 60. Patients with SMA type 1 without treatment do not usually achieve a score greater than or equal to 40 on the CHOP-INTEND scale. Natural history studies show that children with untreated SMA type 1 never gain points on the CHOP-INTEND scale. Three serious adverse events were related or possibly related to the treatment (two patients had elevated hepatic aminotransferases, and one had hydrocephalus)^29^. 

### STR1VE-EU

STR1VE-EU was a phase 3, multicentre, single-arm, open-label trial done at nine sites (hospitals and universities) in Italy (n=4), the UK (n=2), Belgium (n= 2), and France (n=1)^30^. A total of 32 SMA type 1 patients, younger than six months of age, received a single-dose of AVXS-101 and completed the trial. The median age at onasemnogene abeparvovec dosing was 4.1 months. Fourteen (44%) of 32 patients achieved the primary endpoint of functional independent sitting for at least 10 seconds at any visit up to the 18 months of age study visit. Thirty-one (97%) of 32 patients survived free from permanent ventilatory support at 14 months compared with six (26%) of 23 patients in the PNCR natural history cohort. Six (18%) patients had adverse events that were considered serious and related to gene therapy, and increased alanine aminotransferase occurred in nine (27%). One death, unrelated to the study drug, occurred from hypoxic-ischaemic brain damage because of a respiratory tract infection during the study^30^. 

### SPR1NT

This is a phase 3, open-label, single-arm, multicenter, ongoing, single-dose intravenous study of AVXS-101, including pre-symptomatic patients up to six weeks of age with an anticipated development of SMA types 1 or 2 with 2 or 3 copies of the *SMN2* gene. The study included 14 patients with 2 *SMN2* copies, with mean age of 20.6 (8-34) days of life at dosing and mean weight of 3.6 ± 0.39 kg. Fifteen patients with 3 *SMN2* copies were also included, with mean age of 28.7 (9-43) days of life and mean weight of 4.1 ± 0.52 kg at dosing. At the June 2020 visit, all patients were alive and free of permanent ventilation with mean age of 15.6 months (2 copies group) and 15.2 months (3 copies group). In the group of 2 *SMN2* copies, all patients had achieved CHOP-INTEND scores greater than or equal to 50, and 13 (93%) achieved scores greater than or equal to 58. Eleven patients (79%) were able to sit independently for at least 30 seconds at this time of visit. In addition, four patients reached the milestone of independent walking^31^. In the group of patients with 3 *SMN2* copies, 13/15 were able to sit independently for at least 30 seconds, 8/15 reached the primary endpoint that is standing independently and 6/15 patients reached the milestone of walking without assistance^32^. 

## REAL LIFE STUDIES

In a recent publication, Waldrop et al. (2020) reported safety data and baseline outcomes from 21 children (one to 23 months of age) treated with gene therapy in Ohio State, USA^33^. In children ≤ six months, therapy was well tolerated. In this group, elevations in serum transaminases (TGO, TGP) were modest and not associated with elevations in γ glutamyl transferase (GGT). In these cases, administration of prednisolone was uneventful. In older children, increases in serum transaminases and GGT were more frequent and required a higher dose of prednisolone, but all without clinical symptoms. Nineteen of 21 (90%) children experienced an asymptomatic drop in platelets within the first week following treatment, and they recovered without intervention. Of the 19 children who had follow-up data, 11% (n=2) experienced stabilization and 89% (n=17) experienced improvement in motor function^33^. 

Another report published safety and efficacy data from five children who received Nusinersen and gene therapy in combination^34^. Four were receiving Nusinersen before AVXS-101. Nusinersen was continued in three children. Marked elevations of liver enzymes resulted in prolonged treatment with corticosteroids in two patients with hospitalization and liver biopsy in one; milder elevations of liver enzymes were seen in the other two. One patient received, first, gene therapy, and then Nusinersen. No adverse effects were noted. Despite the short follow-up period, the authors considered that the patients improved. They also concluded that long-term use of corticosteroids, as well as monitoring for liver toxicity, may be necessary with gene therapy for SMA^34^.

Weiß C et al. recently published a multicenter study carried out in 18 pediatric neuromuscular centers in Germany and Austria^35^. A total of 76 children with SMA were treated with AVXS-101 (with or without pretreatment with nusinersen) at a mean age of 16.8 months and a mean weight of 9.1 kg. Among 60 patients, 49 had significant improvement on the CHOP-INTEND score (≥4 points) and HFMSE score (≥3 points). Mean CHOP INTEND scores increased significantly in the six months following therapy in children younger than eight months (n=16; mean change 13.8) and children aged between eight and 24 months (n=34; 7.7). No acute complications were reported during infusion of gene therapy, but 56 (74%) patients had treatment-related side-effects. Elevated transaminases significantly increased with age and weight at treatment and this paper reported that six (8%) patients developed acute liver dysfunction^35^.

## GENERAL RECOMMENDATIONS FOR GENE THERAPY IN SMA AND FUTURE TRIALS

Due to the potential for hepatotoxicity observed in clinical trials^36^, onasemnogene aberpavovec for SMA was approved with the recommendation of concomitant use of prophylactic corticosteroids at a dose of 1mg/kg for at least two months after dosing. In addition, weekly monitoring of transaminases and hepatic function in the first month, and every two weeks, in the second month following dosing, is recommended^37^. 

In addition to the long-term efficacy effects, the advent of new post-marketing-reported adverse events has increased caution in the indication of gene therapy in SMA. Recently reported cases of thrombotic microangiopathy, indicated by haemolytic anemia and thrombocytopenia in conjunction with renal failure presented within the first week following administration of gene therapy, have led to changes in recommendations for the treatment and monitoring of these patients^38^ ^,^ ^39^.

Ongoing clinical trials in our country intend to evaluate the outcome of the use of gene therapy for SMA in swallowing (NCT05073133, OFELIA trial). Since 2017, the study with fixed-dose intrathecal administration of onasemnogene aberpavovec (NCT03381729, STRONG study) in older and heavier patients with SMA type 2 has been ongoing, awaiting publication of results of efficacy and extension of the number of subjects in the study.

Finally, the use of this therapy should remain restricted to referral centers with expertise in the management of patients with SMA, and its use in patients older than two years and weighing more than 13.5 kg should ideally be performed in the context of clinical trials^28^.

## ONGOING STUDIES ON OTHER NEUROMUSCULAR DISORDERS AND THEIR POTENTIAL GENE THERAPY

Different neuromuscular disorders of monogenic origin have been the target of pre-clinical and clinical studies using gene therapy. 

In a recent study, two patients with familial ALS and mutations in the gene encoding SOD were treated with a single intrathecal infusion of adeno-associated virus encoding a microRNA targeting *SOD1* ^40^. As a result, *SOD1* protein levels in spinal cord tissue were lower than corresponding levels in untreated patients with *SOD1*-mediated ALS and in healthy controls, showing that gene silencing-therapy could be used as a potential treatment for familial ALS^40^ ^,^ ^41^.

Spinal muscular atrophy with respiratory distress type 1 (SMARD1) is an autosomal recessive motor neuron disease caused by mutations in the *IGHMBP2* gene (11q13). It affects children and currently has no therapeutic options. Recently, adeno-associated virus serotype 9 (AAV9)-mediated gene therapy has been shown to restore protein levels and repair motor function, neuromuscular physiology, while increasing life span in a mouse model^42^. A clinical trial with this strategy is currently ongoing.

Duchenne muscular dystrophy (*DMD*) is an X-linked, muscle-wasting disease affecting males in childhood. The disease is caused by mutations in the *DMD* gene that codes for the subsarcolemmal protein dystrophin^43^. Affected individuals become wheelchair bound by the age of 12 and eventually die in their third decade due to respiratory and cardiac complications. So far, there is no effective treatment for deteriorating muscle function in *DMD* patients. The *DMD* gene is some 11.5 kb long and this large size poses a huge challenge in devising gene transfer therapies into a small viral vector such as AAV. However, observations made in Becker muscular dystrophy patients have suggested that the disease phenotype may be alleviated with a smaller gene construct^44^. In recent studies, recombinant genes encoding multiple variants of micro dystrophin with clinical potential have been generated^45^ ^,^ ^46^, which has led to different clinical trials that are currently ongoing in the United States and Europe, each with a slightly different mini-/micro-dystrophin constructs delivered using AAVs of differing serotypes (rh74, AAV8 and AAV9)^47^. Although the response in motor improvement has been robust in canine models, clinical evidence is lacking that depends on results from ongoing human clinical trials.

Pompe disease (glycogen storage disease II), is a muscle disorder of autosomal recessive origin caused by mutations in the alpha-glucosity acid (*GAA*) gene that has been treated for years with enzyme replacement therapy^48^. However, the disease remains severe and with multisystem involvement mainly in its infantile-onset forms, with severe cardiac and central nervous system involvement. Studies with animal models have already demonstrated the use of gene therapy in reduction of glycogen accumulation in both the myocardium and motorneurons^49^. Several clinical trials are underway with gene replacement therapy with AAV vectors of different serotypes (AAV1, AAV2/8, AAV9) administered intravenously or intramuscularly^50^.

A severe type of congenital myopathy, the X-linked myotubular myopathy (*MTM*), showed great improvements in muscle strength and survival in dogs treated with AAV myotubularin (*MTM1*)-gene therapy^51^. However, in a human clinical trial of *MTM* gene therapy using AAV8, the high-dose group experienced severe hepatotoxicity, which has proven to be lethal in two of these subjects. The exact mechanisms which lead to these adverse events remain unknown, and some hypotheses have been raised on the role of pre-existing antibodies to AAV^52^. Events related to this therapy draw attention to the lethal potential of immunogenic reactions caused by viral vectors.

In conclusion, gene therapy has proven to be an effective therapeutic option in SMA, that serves as a good example of a neuromuscular disease of monogenic origin. However, its long-term effects are still unknown, and some studies even suggest unexpected effects of *SMN1* transgene hyperexpression in animal models, with a potential toxic effect on the dorsal root ganglia^53^ ^,^ ^54^.

Indeed, the management of gene therapy side effects in clinical practice stands as the biggest challenge that clinicians treating patients with neuromuscular disorders will face, and the increasing number of potentially treatable conditions undergoing clinical trials in gene therapy requires an increase in expertise with this new technology therapy.
